# Spatiotemporal regulation of Heterochromatin Protein 1- alpha oligomerization and dynamics in live cells

**DOI:** 10.1038/srep12001

**Published:** 2015-08-04

**Authors:** Elizabeth Hinde, Francesco Cardarelli, Enrico Gratton

**Affiliations:** 1 Laboratory for Fluorescence Dynamics, Department of Biomedical Engineering, University of California, Irvine, USA; 2 Centre for Vascular Research and Australian Centre for NanoMedicine, University of New South Wales, Sydney, Australia; 3 Center for Nanotechnology Innovation @NEST, Istituto Italiano di Tecnologia, Piazza San Silvestro 12 - 56127 Pisa, Italy

## Abstract

Heterochromatin protein 1 (HP1) is a central factor in establishing and maintaining the heterochromatin state. As consequence of playing a structural role in heterochromatin, HP1 proteins can have both an activating as well as repressive function in gene expression. Here we probe how oligomerisation of the HP1-α isoform modulates interaction with chromatin, by spatially resolved fluorescence correlation spectroscopy (FCS). We find from fluctuation analysis of HP1-α dynamics that this isoform exists as a dimer around the periphery of heterochromatin foci and these foci locally rotate with characteristic turn rates that range from 5–100ms. From inhibition of HP1-α homo-oligomerization we find the slow turn rates (20–100 ms) are dimer dependent. From treatment with drugs that disrupt or promote chromatin compaction, we find that HP1-α dimers spatially redistribute to favor fast (5–10 ms) or slow (20–100 ms) turn rates. Collectively our results demonstrate HP1-α oligomerization is critical to the maintenance of heterochromatin and the tunable dynamics of this HP1 isoform.

Dynamic changes in higher order chromatin structure modulate the accessibility of DNA for proteins involved in transcription, DNA repair and replication events^1^,^2^,^3^ as well as for inert molecules^4^,^5^,^6^,^7^. More in detail, by recruitment of histones and other chromosomal proteins to the DNA template and modulation of their binding affinities based on the genes encoded, chromatin is organized into two different compaction states: the denser and transcriptionally repressed *hetero*chromatin and the more open and biologically active *eu*chromatin^8^,^9^. A chromosomal protein central to establishing and maintaining the heterochromatin state is heterochromatin protein 1 (HP1). Biochemical and structural characterization of HP1 proteins have helped provide molecular explanations for their roles in heterochromatin. These studies have identified multiple domains within HP1 proteins: the chromodomain (CD), which specifically recognizes the di- and trimethylation of lysine 9 on histone H3 (H3K9me2/3) mark; the chromoshadow domain (CSD), which forms a dimerization interface that recruits specific ligands; and a connecting hinge region, which interacts with nucleic acids^10^,^11^,^12^,^13^,^14^. There are three HP1 isoforms (α, β, γ) that are conserved across most eukaryotes and although all three are structurally similar, they differ in terms of their specific localization within the nucleus^15^. The two dominant isoforms, HP1-α and HP1-β, are primarily associated with heterochromatin whereas the Hp1-γ isoform localizes to a larger extent to euchromatin^16^,^17^. Thus, depending on the particular isoform, HP1 proteins can have both an activating as well as repressive function in gene expression^18^. As silencing factors they condense chromatin, thus preventing efficient transcription initiation; as activators, they create a particular chromatin environment conducive to transcription^8^.

The spatiotemporal dynamics of HP1 are thus critical to the regulation of several nuclear functions and as a result HP1 has become one of the most intensely studied proteins in the chromatin field^1^. Experiments based on fluorescence correlation spectroscopy (FCS) and fluorescence recovery after photobleaching (FRAP) have revealed the HP1 proteins to adopt different dynamics in regions of heterochromatin versus euchromatin due to different binding affinities^1^,^19^,^20^,^21^. It is reported that the nuclear pool of HP1 can be separated into three fractions: *i*) a highly mobile fraction, *ii*) a less mobile, transiently binding fraction and *iii*) a minor fraction that is quite immobile^1^. The percentage population of each fraction differs between regions of heterochromatin to euchromatin depending on the isoform. Although these studies detected the ‘tunable’ dynamics of the different HP1 isoforms, it remains unclear as to how HP1 proteins can elicit the distinct modes of interaction identified, and differentially between euchromatin and heterochromatin. From *in vitro* studies it is thought that the formation of HP1α homo-dimers change its interaction^10^,^22^ and induce the nucleation of a higher order chromatin structure that is incompatible with transcription^8^,^20^,^23^. Direct measurement of such dynamics *in vivo* however is challenging as high spatiotemporal resolution and high sensitivity is required.

In previous works we measured chromatin dynamics *indirectly* by pair correlation function analysis of enhanced green fluorescent protein (EGFP) molecular flow through regions of heterochromatin and euchromatin^5^,^6^,^7^,^24^. This is done by a fast scan along a line that includes chromatin. The pair correlation algorithm then calculates the spatial cross-correlation functions for all the pixel distances along the line: if there is molecular flow in the pair of pixels measured along the line, then there will be a positive cross-correlation among the pixel pair with some time delay that depends on the time a molecule takes to flow from one pixel to the other^25^,^26^. Here we adapt this approach to measure higher order chromatin structure and dynamics *directly*, by fluctuation analysis of the fluorescently labeled HP1-α isoform. More in detail, we preliminarily probed, for the first time, the actual existence and precise localization of HP1α homo-dimers in the nuclei of live cells by the Number and Brightness (N&B) analysis^27^. We found that the HP1-α isoform exists as a dimer around the periphery of heterochromatin foci. Then, by pair correlation analysis of HP1-α molecular diffusivity we tested the possible correlation between HP1-α dimerization and chromatin accessibility at the level of heterochromatin foci. We found that these foci macroscopically rotate with characteristic turn rates and from a single point mutation that the slow turn rates are HP1-α dimer dependent. Treatment with drugs that are known to disrupt or promote chromatin compaction, reorganized the localization of HP1-α dimers into a diffuse or highly-localized population, respectively, and these configurations resulted in long or short time scale turn rates being favored for heterochromatin foci rotation, respectively. Collectively, these results suggest that homo-oligomerization of HP1-α is critical to the maintenance of heterochromatin and the ability of this protein to elicit a distinct mode of interaction with transcriptionally active euchromatin.

## Results

**Figure 1:**
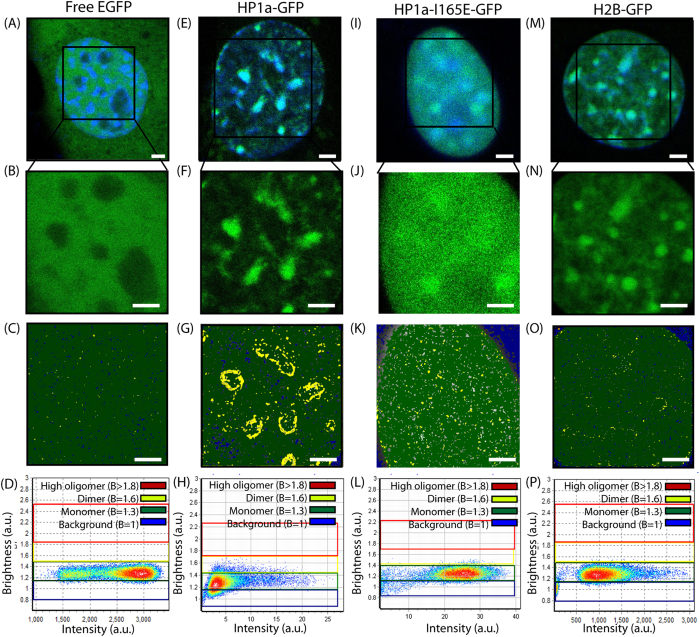
Number and brightness analysis of HP1 alpha oligomerization. (**A**) Intensity image of a NIH3T3 nucleus expressing free EGFP with the DNA stained by Hoechst 33342. (**B**) Region of interest from (**A**) selected for brightness analysis in the EGFP channel. (**C**) Region of interest in (**B**) pseudo-colored according to the brightness distribution of free EGFP in (**D**). As can be seen free EGFP only exists as a monomer throughout the nucleus. (**E**) Intensity image of a NIH3T3 nucleus expressing HP1-α-EGFP with the DNA stained by Hoechst 33342. (**F**) Region of interest from (**E**) selected for brightness analysis in the HP1-α-EGFP channel. (**G**) Region of interest in (**F**) pseudo-colored according to the brightness distribution of HP1α in (**H**). As can be seen dimers are located around the periphery of the heterochromatin foci. (**I**) Intensity image of a NIH3T3 nucleus expressing HP1-α-I165E-EGFP with the DNA stained by Hoechst 33342. (**J**) Region of interest from (**I**) selected for brightness analysis in the HP1-α-I165E-EGFP channel. (**K**) Region of interest in (**J**) pseudo-colored according to the brightness distribution of HP1-α-I165E-EGFP in (**L**). As can be seen HP1-α-I165E-EGFP is monomeric throughout the nucleus even at the periphery of heterochromatin foci. (**M**) Intensity image of a NIH3T3 nucleus expressing free H2B-EGFP with the DNA stained by Hoechst 33342. (**N**) Region of interest from (**M**) selected for brightness analysis in the H2B-EGFP channel. (**O**) Region of interest in (**N**) pseudo-colored according to the brightness distribution of H2B in (**P**). As can be seen H2B is monomeric throughout the nucleus even at the periphery of heterochromatin foci.

Here we use NIH3T3 cells transiently transfected with HP1α-EGFP and stained with Hoechst 33342 (as a reference of DNA) to measure the spatiotemporal regulation of HP1-α oligomerization within the nuclear compartment and how this modulates HP1-α dynamics. To investigate HP1-α oligomerization we use the well-established N&B approach^27^. With this method we first established the brightness of monomeric EGFP so that the oligomerization state(s) of HP1-α-EGFP can be eventually derived from an increase of the molecular brightness. To determine the monomer value we performed brightness experiments on cells transiently transfected with monomeric EGFP and stained with Hoechst 33342 to ensure that pixels containing the DNA-stain did not alter the detected brightness of pixels in the EGFP channel. By visual inspection, we selected a transfected cell (Fig. 1A) and then digitally zoomed in on a plane within the nucleus (Fig. 1B) to acquire a time series of 100 frames in the EGFP channel (the experimental settings are detailed in the Materials and Methods section). We then carried out the N&B analysis of the recorded fluorescence intensity fluctuations in each pixel of the selected plane which gives the molecular brightness (ε) of monomeric EGFP. From construction of the measured apparent brightness (B_apparent_ = ε + 1) in each pixel of the image into a brightness map (Fig. 1C, is pseudo-coloured according to the green cursor placed over the measured brightness distribution in Fig. 1D), we found EGFP to have an apparent brightness distribution centered at ε = 1.3. Given that this value includes the molecular brightness of background (B_background_ = 1), the molecular brightness of monomeric EGFP is thus 0.3 counts/dwelltime/s. From this value we can extrapolate that a HP1-α dimer would have an apparent brightness of 1.60 (given that B_dimer_ = (2 × 0.3) = 0.6), a HP1-α trimer would have an apparent brightness of 1.90 (given that B_trimer_ = (3 × 0.3) = 0.9) and so on.

Based on this calibration we then performed the N&B experiment in a cell transiently transfected with HP1-α-EGFP and stained with Hoechst 33342 (Fig. 1E), to see where and to what degree HP1α oligomerizes in the nucleus. From N&B analysis in each pixel of the selected plane (Fig. 1F) we found from the derived brightness map (Fig. 1G, pseudo-colored according to oligomerization state; palette defined in Fig. 1H) that the HP1-α protein forms a dimer around the edges of the heterochromatin foci (yellow pixels) and is monomeric throughout the rest of the nucleoplasm (green pixels). This is in keeping with the *in vitro* study that reported HP1-α dimers to induce the nucleation of a higher order chromatin structure that is incompatible with transcription. To prove that the detected increase of molecular brightness observed at the periphery of heterochromatin foci is in fact due to HP1-α-EGFP oligomerization and not solely HP1-α binding interaction, we then performed the same type of experiment in a cell transiently transfected with HP1-α-I165E-EGFP (Fig. 1I), since this mutant is unable to form dimers^28^. From N&B analysis in each pixel of the selected plane (Fig. 1J) we found from the derived brightness map (Fig. 1K, pseudo-colored according to oligomerization state; palette defined in Fig. 1L) that HP1-α-I165E-EGFP does not yield a brightness value higher than the monomeric state throughout the entire nucleus and therefore the higher molecular brightness detected for HP1-α-EGFP is due to dimerization. To prove that the peripheral localization of HP1-α-EGFP oligomerization is not an artefact of heterochromatin foci movement, we then performed the same experiment in a cell transiently transfected with H2B-EGFP and stained with Hoechst 33342 (Fig. 1I), since this histone is stably bound to the heterochromatin on the timescale of the experiment^29^. From N&B analysis in each pixel of the selected plane (Fig. 1M) we found from the derived brightness map (Fig. 1N, pseudo-colored according to oligomerization state; palette defined in Fig. 1M) that H2B does not yield a brightness value higher than the monomeric state throughout the entire nucleus. Also, we excluded possible artefacts due to putative Hoechst influence on chromatin organization. As shown in Supplementary Figure 1, in fact, cells co-transfected with HP1-α-EGFP and H2B-mCherry, but in absence of Hoechst, yield similar N&B results.

**Figure 2:**
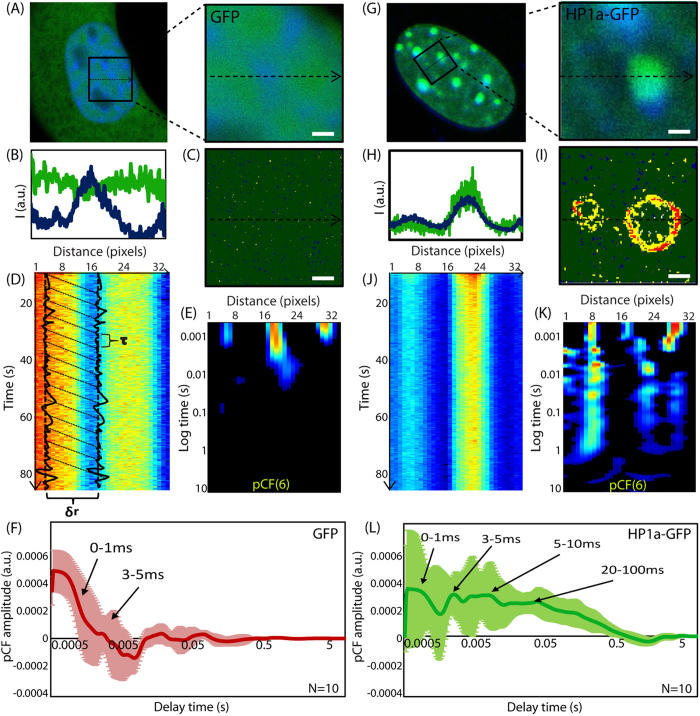
Pair correlation analysis of HP1 alpha molecular flow with respect to heterochromatin foci. N&B analysis of HP1-α oligomerization. (**A**) Region of interest selected for pair correlation analysis in a NIH3T3 nucleus expressing free EGFP with the DNA stained by Hoechst 33342. (**B**) Intensity profile of free EGFP overlaid with the intensity profile of Hoechst 33342 along the selected region of interest reveals a heterochromatin region between pixels 8–16. (**C**) Region of interest in (**A**) pseudo-colored according to the brightness distribution of free EGFP (green = monomer, yellow = dimer, red = higher order oligomer). (**D**) Line scan acquired along the region of interest in the EGFP channel reveals an exclusion from the heterochromatin region. (**E**) Pair correlation analysis of free EGFP molecular flow with respect to a compact chromatin density region. (**F**) Average pair correlation profile for EGFP molecular flow in regions of heterochromatin foci (N = 10 cells) reveals molecular mobility on a time scale of 1–5 ms. (**G**) Region of interest selected for pair correlation analysis in a NIH3T3 nucleus expressing HP1-α-EGFP. (**H**) Intensity profile of HP1-α-EGFP overlaid with the intensity profile of Hoechst 33342 along the selected region of interest reveals a heterochromatin region between pixels 4–12 and 20–28. (**I**) Region of interest in (**G**) pseudo-colored according to the brightness distribution of HP1-α-EGFP (green = monomer, yellow = dimer, red = higher order oligomer). (**J**) Line scan acquired along the region of interest in the HP1-α-EGFP channel reveals a co-localisation with the heterochromatin regions. (**K**) Pair correlation analysis of HP1-α-EGFP molecular flow with respect to heterochromatin foci reveals multiple bands of correlation at the edges of the foci. pCF(6) in (**E**) and (**G**) indicates that pair correlation function is calculated at the distance of 6 pixels along the scanned line (see also Methods for further details). (**L**) Average pair correlation profile for HP1-α-EGFP molecular flow in regions of heterochromatin foci (N = 10 cells) molecular mobility on a timescale of 1–5 ms and then re-appearance due to a potential rotation from 5–10 ms and 20–100 ms.

One important question is whether there is a different spatio-temporal dynamics of the homo-dimers with respect to the monomers that could indicate that homodimers could be involved in chromatin compaction? We then decided to investigate if and how the HP1-α homo-dimers change the spatiotemporal dynamics of HP1-α with respect to heterochromatin foci by pair correlation analysis of HP1-α-EGFP molecular flow. We need to first understand how heterochromatin foci affect passive diffusion of an inert molecule such as free EGFP as evidenced in the pair-correlation carpet, so that any deviation from the pair correlation carpet obtained for EGFP can be attributed to HP1α interaction. Thus as can be seen in Fig. 2A we first measured an NIH3T3 cell transiently transfected with EGFP and stained with Hoechst 33342, then based on the DNA staining, we selected a line scan within this cell’s nucleus that traversed a highly compact chromatin region. This can be seen from the intensity profile of Hoechst 33342 along the selected line scan (Fig. 2B). Brightness analysis of free EGFP in the plane of the selected line scan (Fig. 2C), shows EGFP to be monomeric irrespective of the traversed heterochromatin region. If we then scan the selected line rapidly as a function of time in the EGFP channel (Fig. 2D) we see that free EGFP is slightly excluded from the heterochromatin region located between pixels 10–20. From pair correlation analysis of EGFP molecular flow with respect to this high density environment (Fig. 2E) we show that there is no molecular flow (exchange) between these two environments, although EGFP diffusion is detected inside and outside of this region. From analysis of N = 10 heterochromatin foci, we find free EGFP to have a molecular mobility of 1–5 ms in this compact environment (Fig. 2F).

If we then compare this baseline diffusive behavior of free EGFP with respect to heterochromatin, with the diffusive behavior of an EGFP-tagged heterochromatin specific protein such as HP1-α, we find a very different result. As can be seen in Fig. 2G, if we select a region of interest that traverses heterochromatin foci we see that HP1-α co-localizes with the Hoechst 33342 stain (Fig. 2H) and there are HP1-α dimer located along the periphery (Fig. 2I). If we then scan this region of interest rapidly as a function of time (Fig. 2J) and perform pair correlation analysis on HP1-α-EGFP molecular flow we find that in those columns which line up with the edges of the heterochromatin foci there are multiple bands of positive correlation occurring on discrete timescales (Fig. 2K). In particular, from analysis of N = 10 heterochromatin foci we detect an initial decay from 1–5 ms that is in agreement with free EGFP molecular mobility and then addition discrete peaks of correlation on a timescale of 5–10 ms and 20–100 ms (Fig. 2L). This indicates that the HP1-α molecules reappear at the same position by a non-random mechanism that we argue to be compatible with an overall local rotation of the heterochromatin foci.

**Figure 3:**
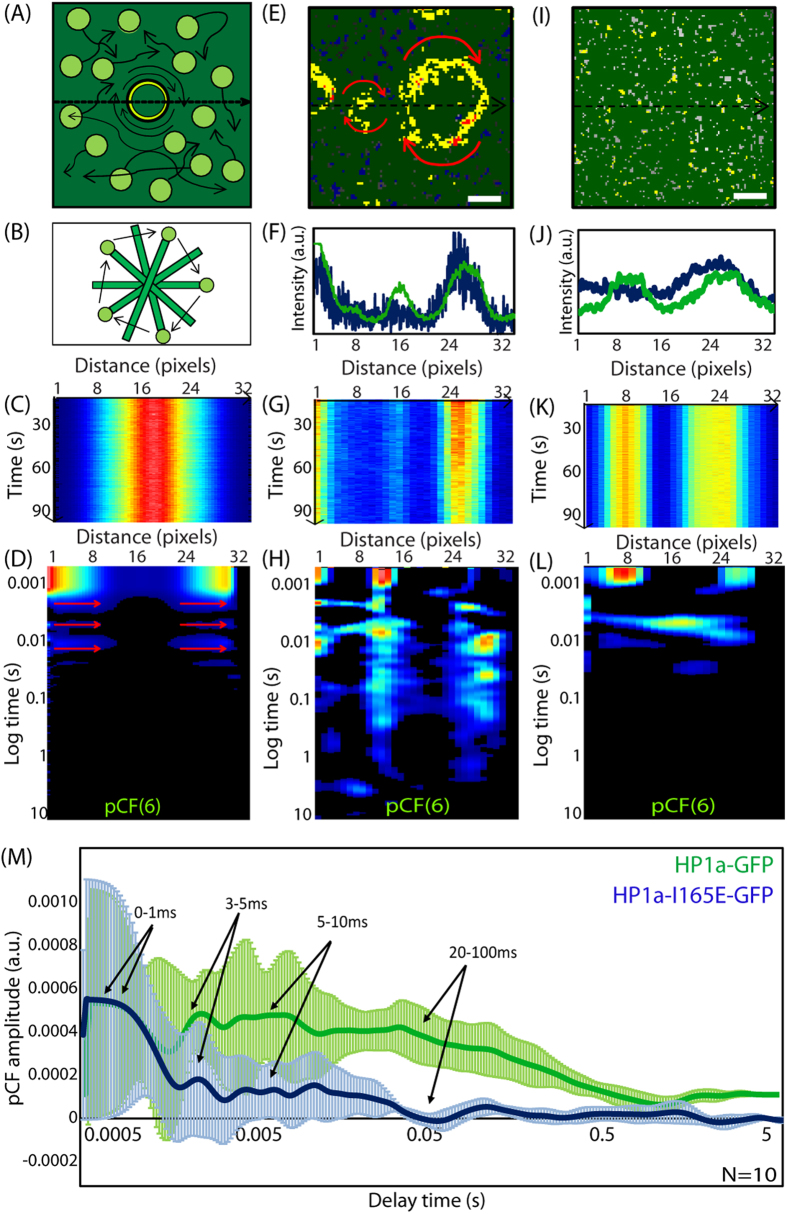
HP1-α heterochromatin foci rotate at different turn rates that are dependent on dimerization. (**A**) *In vivo* data suggests HP1-α to be bound to the edges of the heterochromatin foci as a dimer and foci to rotate with a characteristic time. (**B**) We simulate the rotating heterochromatin foci with a dimer periphery as a rotating stick with a fluorescent molecule on one end. (**C**) A simulated line scan acquired across the rotating stick results in a ‘heterochromatin foci’ between pixels 12–20. (**D**) Pair correlation analysis along the simulation line scan results in the multiple bands of correlation only at the ends of the simulated heterochromatin foci. (**E**) Brightness analysis of HP1-α dimerization around periphery of heterochromatin foci. (**F**) Intensity profile of free EGFP overlaid with the intensity profile of Hoechst 33342. (**G**) Line scan acquired along the region of interest in the HP1-α-EGFP channel reveals a co-localisation with the heterochromatin regions. (**H**) Pair correlation analysis of HP1-α-EGFP molecular flow reveals multiple bands of correlation at the edges of the foci. (**I**) Brightness analysis of HP1-α-I165E reveals loss of dimerization around periphery of heterochromatin foci. (**J**) Intensity profile of HP1-α-I165E-EGFP overlaid with the intensity profile of Hoechst 33342. (**K**) Line scan acquired along the region of interest in the HP1-α-I165E-EGFP channel reveals a co-localisation with the heterochromatin regions. (**L**) Pair correlation analysis of HP1-α-I165E-EGFP molecular flow reveals a loss of the long time scale bands of correlation at the edges of the foci. (**M**) Overlay of the average pair correlation profile for HP1-α-EGFP molecular flow (N = 10 cells) and HP1-α-I165E-EGFP molecular flow (N = 10 cells) in regions of heterochromatin foci, reveals the re-appearance of molecules on a timescale of 20–100 ms to be lost upon inhibition of dimerization.

To test the hypothesis that the periodic correlation pattern observed in the pair-correlation carpet could be due to local rotation of a relatively rigid structure to which the HP1-α is attached, we simulated the rotating heterochromatin foci fluorescently tagged with HP1-α-EGFP as a rotating stick with a fluorescent molecule on one end (Fig. 3A). We then simulated a line scan across this rotating structure (Fig. 3B) and constructed the lines into an intensity carpet (Fig. 3C). As can be seen in Fig. 3C this simulation produced an intensity carpet that is similar to what would be observed for a single heterochromatin region in the middle of the line scan. We then carried out pair correlation analysis of this rotating fluorescent structure at a distance which catches molecular flow along the periphery (Fig. 3D), to see if a single speed rotation could produce correlations with similar features to that observed in Fig. 2K. As can be seen in Fig. 3D, the simulation reproduces the discrete and intermittent bands of correlation observed in real experiments on HP1-α (Fig. 3E–H). However we find that the *in vivo* data acquired across different HP1-α-EGFP tagged heterochromatin foci is more complex and in contrast to the simulation, must be the result of more than one characteristic turn rate for different heterochromatin foci on the timescale of the experiment. The dimerization around the periphery of the heterochromatin foci appears to be important for determining the long rotation times, given that the multiple banded pattern of correlation (see red arrows in Fig. 3H) is only observed in the presence of the HP1-α dimer.

To test if the multiple bands of correlation observed at long time scales is dependent on HP1-α dimerization, we then carried out pair correlation analysis of the HP1-α-I165E mutant, which as detected from a brightness analysis in Fig. 1M–P, cannot form dimers. As can be seen in Fig. 3I, the HP1-α-I165E mutant is monomeric throughout the nucleoplasm, even in regions where there are heterochromatin foci as detected by overlaying the HP1-α-I165E-EGFP fluorescence intensity with the Hoechst 33342 stain (Fig. 3J). If we scan a line rapidly across the detected heterochromatin foci in only the EGFP channel (Fig. 3K) and then carry out pair correlation analysis on HP1-α-I165E-EGFP molecular flow (Fig. 3L), we find indeed that the long time scale bands of correlation observed in Fig. 3H are absent and only a short time scale bands of correlation remain. From analysis of N = 10 wild type heterochromatin foci versus N = 10 heterochromatin foci formed from the HP1-α I165E mutant (Fig. 3M), we find that the HP1-α dimers are critical to the stabilization of slow turn rates (20–100 ms), given that the HP1-α-I165E monomers are on average not detected in a given pixel beyond 20 ms. The role of this dimer stabilized turn rate remains elusive, although it must to be critical to maintaining a condensed heterochromatin organization given that the foci of the HP1-α I165E mutant, as depicted in Fig. 1I-G, appear more diffuse than the wild type foci Fig. 1A,B.

**Figure 4:**
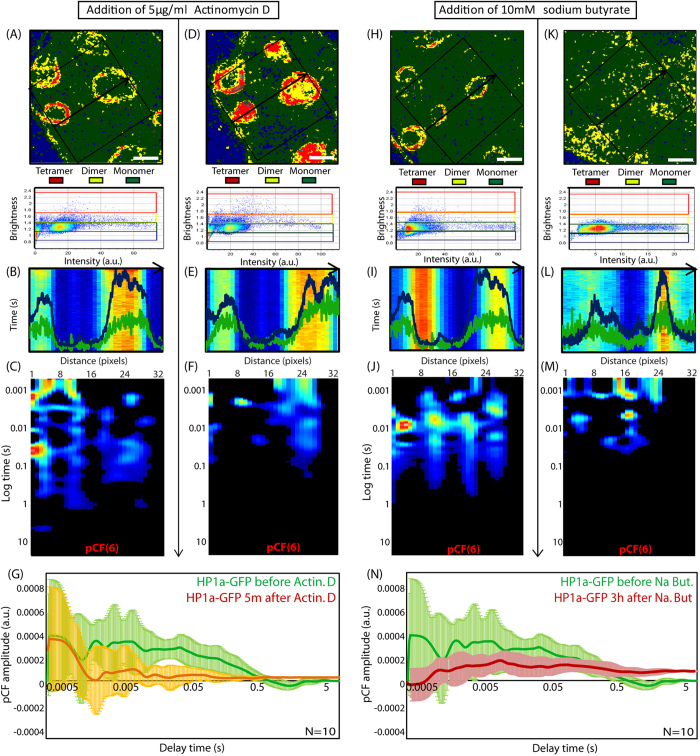
Induction of chromatin compaction or loosening by drug treatment disrupts HP1-α oligomerization and molecular flow in both cases. (**A**) Brightness analysis of HP1-α dimerization around periphery of heterochromatin foci. (**B**) Intensity profile of free EGFP and Hoechst 33342 superimposed over the line scan acquired across the heterochromatin foci in (**A**). (**C**) Pair correlation analysis of HP1-α-EGFP molecular flow reveals the multiple bands of correlation observed in Figs 2 and 3, at the edges of the foci. (**D**) Brightness analysis of HP1-α after treatment with Actinomycin D reveals HP1α to further oligomerize into a tetrameric form around the periphery of the heterochromatin foci. (**E**) Intensity profile of free EGFP and Hoechst 33342 superimposed over the line scan acquired across the heterochromatin foci in (**D**). (**F**) Pair correlation analysis of HP1-α-EGFP molecular flow after Actinomycin D treatment reveals the multiple bands of correlation observed in (**C**) to be disrupted. (**G**) Overlay of the average pair correlation profile for HP1-α-EGFP molecular flow before and after treatment with Actinomycrin D (N = 10 heterochromatin foci) reveals the re-appearance of molecules on a time scale of 20–100 ms to be inhibited. (**H**) Brightness analysis of HP1-α dimerization around periphery of heterochromatin foci. (**I**) Intensity profile of free EGFP and Hoechst 33342 superimposed over the line scan acquired across the heterochromatin foci in (**H**). (**J**) Pair correlation analysis of HP1-α-EGFP molecular flow reveals the multiple bands of correlation observed in Figs 2 and 3, at the edges of the foci. (**K**) Brightness analysis of HP1-α after treatment with Sodium Butyrate reveals HP1α oligomerization around the heterochromatin foci to be inhibited. (**L**) Intensity profile of free EGFP and Hoechst 33342 superimposed over the line scan acquired across the heterochromatin foci in (**K**). (**M**) Pair correlation analysis of HP1-α-EGFP molecular flow after treatment with sodium butyrate reveals the multiple bands of correlation in (**L**) to be disrupted. (**N**) Overlay of the average pair correlation profile for HP1-α-EGFP molecular flow before and after treatment with Sodium Butyrate (N = 10 heterochromatin foci) reveals the re-appearance of molecules on a time scale of 5–10 ms to be inhibited.

To further investigate this we induced a compacted chromatin structure which inihibits DNA template accessibility by treatment with Actinomycin D (Fig. 4A–F) and found that the HP1-α oligomerization along the periphery of the heterochromatin foci is promoted to a tetrameric state (Fig. 4D) and the rotation of heterochromatin foci on long timescales stops. In particular, from analysis of N = 10 compacted heterochromatin foci, we detect the peaks of correlation on a timescale of 20–100 ms to be significantly inhibited (Fig. 2G). If in contrast, however, we loosen the chromatin structure to facilitate DNA template accessibility by treatment with sodium butyrate (Fig. 4H–M), we find the dimer along the periphery to disassemble and the rotation on short timescales to stop. In particular, from analysis of N = 10 de-condensed heterochromatin foci, we detect the peaks of correlation on a timescale of 1–5 ms to be significantly inhibited (Fig. 4N). Thus it seems there is a strong interplay between HP1-α oligomeric state and foci dynamics that is responsive to the compaction state of the chromatin.

## Discussion

Proper understanding of how heterochromatin is established and maintained will ultimately enhance our understanding of stable gene repression. Here, we detected for the first time *in vivo* HP1-α dimer formation around the periphery of heterochromatin foci in live cells. In agreement with previous *in vitro* studies^10^,^22^,^23^, we also found the HP1-α homo-dimers to modulate HP1-α spatiotemporal dynamics. In particular we found that heterochromatin foci with the periphery decorated by HP1-α dimers rotate with different characteristic rates and from a single point mutation that inhibits HP1-α homo-oligomerization, this local rotation is dimer dependent and critical to the maintenance of heterochromatin structure. It is reported *in vitro* that the HP1-α homo-dimers nucleate the formation of a higher order structure that is incompatible with transcription. Here we support this observation in live cells by loosening the chromatin into a de-condensed transcriptionally accessible state and detecting the loss of dimerization. In addition we found that if we instead promote chromatin compaction into an even more transcriptionally inaccessible state, HP1-α oligomerization is promoted into a tetrameric state, a higher order oligomer that, to our knowledge, is observed here for the first time in live cells (and was never observed before *in vitro*). In both instances the rotation of heterochromatin foci is modulated, to favor either slow or fast turn rates, and thus it appears that the tunable dynamics of HP1-α is achieved by changes in its oligomerization state. In general, two major findings here are somewhat surprising and deserve a more detailed discussion. First, the HP1-α oligomers (dimers or tetramers) are found only at the heterochromatin periphery (irrespective of the size of the heterochromatin focus), while the monomeric form is found at the heterochromatin center and throughout the nucleoplasm. This in turn suggests that a precise spatial organization of HP1-α oligomers is crucial for the regulation of heterochromatin organization. We may speculate that HP1-α dimers at the periphery of heterochromatin foci form a dimerization interface that recruits specific ligands, presumably involved in heterochromatin spreading, although the mechanism of spreading is still to be clarified^30^,^31^. In this context, HP1-α tetramerization may represent a molecular switch to stop heterochromatin spreading progression, and all the related processes. Second, the periodic structure of the pair-correlation carpet is compatible with the rotation of heterochromatin foci, as demonstrated by simulations. Although uncommon, this pattern of correlation may reflect ongoing active structural rearrangements at the level of heterochromatin foci. As stated above, our data suggest that HP1-α may play an active role in promoting the rotation effect. In this regard, strict modulation of rotation dynamics may be needed more for HP1-α than for other homologs because the assembly and disassembly of HP1-α needs to be coordinately regulated over large stretches of the genome^31^. At the same time, the presence of multiple correlation bands, ranging from few milliseconds to hundreds of milliseconds, suggests a kind of flexibility of this process, maybe reflecting the well-known structural and functional versatility of HP1-α^31^.

To conclude, we may speculate that an intranuclear heterochromatin target area of particular interest for future investigations might be *pericentric* heterochromatin, a major repressive chromatin domain, mainly composed of major and minor satellite repeats (easily visualized as DAPI-dense spots in interphase nuclei). Different functions have been assigned to this repressive chromatin locus, the most commonly reported being its participation in kinetochore attachment in mitosis and subsequent chromosome segregation. However, pericentric heterochromatin seems to play also important roles in organizing the repressive compartments of the nucleus, probably by recruitment of silenced genes to the pericentric region, as shown for imprinting or allelic exclusion during B cell development^32^. Interestingly, pericentric heterochromatin is enriched for epigenetic modifications which correlate with gene repression. More in detail, the major histone lysine methylation marks at pericentric heterochromatin are H3K9me3 and H4K20me3. Suv39h1 and Suv39h2 enzymes establish H3K9me3 while Suv4-20h1 and Suv4-20h2 are the major enzymes to induce H4K20me3^33^. A sequential pathway connects these two histone methylation systems: H3K9me3 is established first, providing a binding platform for HP1 proteins; HP1 isoforms in turn recruit Suv4-20h enzymes which establish H4K20me3. Consistent with these findings, H4K20me3 is lost in either Suv39h double null or HP1 mutants. Another major epigenetic modification of pericentric heterochromatin is DNA methylation. There is evidence for a complex interplay of Suv39h enzymes (and H3K9me3) with DNA methyltransferases, although this is currently not understood at a mechanistic level)^34^. In this complex picture, we are prompted to argue that the HP1-α spatiotemporal dynamics and oligomerization state regulation observed here must play a pivotal role in directing/modulating HP1-α interactions with its proteic partners, and must be taken into account in future studies. More in general, we believe that, among others, a thorough mechanistic insight into epigenetic programming at pericentric heterochromatin will enhance our understanding of many important cellular processes ranging from cancer to aging.

## Methods

### Sample preparation

NIH3T3 cells were grown in high glucose medium from Invitrogen, supplemented with 10% Fetal Bovine Serum, 5 ml of Pen-Strep and HEPES at 37°C and in 5% CO_2_. Freshly split cells were plated onto 35-mm glass bottom dishes coated with fibronectin and then, after twenty four hours, transiently transfected with EGFP, H2B-EGFP (Plasmid #11680 purchased from Addgene), HP1α-EGFP (Plasmid #17652 purchased from Addgene) or HP1α-I165E-EGFP (kindly provided by Professor Lori Wallrath). Freshly split cells were plated onto MatTek 35-mm glass bottom dishes coated with 3 μg/mL fibronectin and then transiently transfected with one of the EGFP tagged plasmids using Lipofectamine 2000 according to manufacturer’s protocol. Induction of chromatin compaction was carried out by treating the NIH3T3 cells transiently transfected with HP1α with Actinomycin D (5 μg/ml, a concentration known to stop class III transcription) for 5–10 minutes. Induction of chromatin loosening was carried out by treating the NIH3T3 cells transiently transfected with HP1α with sodium butyrate (10 mM, a concentration known to inhibit Histone Deacetylase, HDAC) for 1–4 hours^35^.

### Microscopy

The microscopy measurements were performed on a Zeiss LSM710 Quasar laser scanning microscope, using a 40X water immersion objective 1.2 N.A. (Zeiss, Germany). EGFP was excited with the 488 nm emission line of an Argon laser. Hoechst 33342 was excited with the 405 nm emission line of a diode laser. EGFP and Hoechst were measured sequentially using the 510–560 nm and 410–490 nm emission ranges, respectively. For each channel the pinhole was set to 1 Airy Unit. Image acquisition for Number and Brightness analysis involved selecting a region of interest within a NIH3T3 cell nucleus at an electronic zoom that resulted in a pixel size of 50–100 nm for a 256 × 256 pixel frame size. A time series of 100 frames was then collected in the EGFP channel at this zoom, with the pixel dwell time set to 12.61 μs/pixel, which resulted in a line time of 7.56 ms and a frame time of 1.15 s. Calibration of the monomeric brightness of the EGFP based constructs was performed by measurement of cells transfected with free EGFP under identical experimental conditions. Line scan acquisition for pair correlation analysis involved selecting a 3 μm line within the NB acquisition ROI that traversed heterochromatin foci (as indicated by the Hoechst 33342 or HP1α fluorescence intensity). The selected line was then rapidly scanned 2 × 10^5^ times in the EGFP channel at maximum speed (pixel dwell time 6.3 μs, line time 0.472 ms), with fluorescence being sampled every 100 nm (32 pixels to a line). The average laser power at the sample for both frame and line scan acquisitions was maintained at the 1–2 mW. The data acquired were processed by the SimFCS software developed at the Laboratory for Fluorescence Dynamics (www.lfd.uci.edu).

### Number and Brightness analysis

Calculation of the Number and Brightness of molecules in each pixel of an image was performed using the SimFCS software available at www.lfd.uci.edu, as described in previously published papers^27^,^36^. Briefly in each pixel of a time series of frames we have an intensity fluctuation that has an average intensity ‹k› (first moment) and a variance σ^2^ (second moment). These two properties describe the apparent number (N) and brightness (B) of the molecules that give rise to the intensity fluctuation, according to equations 1 and 2:


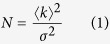



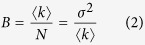


The true molecular brightness (ε) of the molecules that give rise to the measured apparent brightness (B) in the case of a photon counting detector are related according to equation 3:


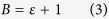


where 1 is the brightness contribution of the detector given that the photon counting detector variance (σ^2^_detector_) should equal the average intensity of the detector noise (‹k›_detector_). In the case of an analogue detector (as was used in the results presented here) this is not true due to characteristics of the analog amplifier and the settings of the analog-to-digital converter. Thus the detector’s brightness contribution needs to be accounted for by a term we call the S factor, which returns the background brightness to 1 so that the molecular brightness of the molecules can be extracted. By analysis of pixels that did not contain fluorescence from the sample, we found the brightness of the background to be between 1.3–1.6 depending on the laser power required. Taking into account this S factor we then calibrated the monomeric brightness of free EGFP so that in the instance oligomerization was observed when tagged to H2B or HP1α we could extract the stoichiometry of the oligomer. The effect of cell movement was subtracted by the moving average algorithm, as previously described^27^.

### Pair Correlation analysis

Calculation of the pair-correlation functions was done using the SimFCS software developed at the Laboratory for Fluorescence Dynamics (www.lfd.uci.edu), as described in the Supplementary material and previously published papers^5^,^6^,^7^,^25^. Intensity data are presented by using a carpet representation in which the *x*-coordinate corresponds to the point along the line (pixels) and the *y*-coordinate corresponds to the time of acquisition. The pair correlation function (pCF(pixel distance)) is displayed in pseudo colors in an image in which the *x*-coordinate corresponds to the point along the line and the *y*-coordinate corresponds to the autocorrelation time in a log scale. The distances at which pCF analysis was carried out were not fixed across all experiments, but instead determined on an individual basis by the chromatin density variation along each line measured. In general a distance of 4–8 pixels (which corresponds to 400–800 nm) was employed. In general for each experiment, 2 × 10^5^ consecutive lines (with no intervals between lines) were acquired. Time regions within each experiment (~6.4 × 10^4^ lines, corresponding to ~30 s) with no average change in fluorescence intensity (e.g. due to photobleaching) were then selected for the correlation analysis, as also described elsewhere^6^,^7^.

## Additional Information

**How to cite this article**: Hinde, E. *et al*. Spatiotemporal regulation of Heterochromatin Protein 1- alpha oligomerization and dynamics in live cells. *Sci. Rep.* **5**, 12001; doi: 10.1038/srep12001 (2015).

## Supplementary Material

Supplementary Information
