# Supplementary material for: Spatiotemporal regulation of Heterochromatin Protein 1- alpha oligomerization and dynamics in live cells
Source: Sci Rep. 2015 Aug 4;5:12001. doi: 10.1038/srep12001 (PMC4523856; doi:10.1038/srep12001)
Supplement: Supplementary Information [file srep12001-s1.pdf]

**Supporting Material**

Spatiotemporal regulation of Heterochromatin Protein 1- alpha oligomerization and dynamics in live cells

Elizabeth Hinde[a,b], Francesco Cardarelli[a,c], Enrico Gratton[a*].

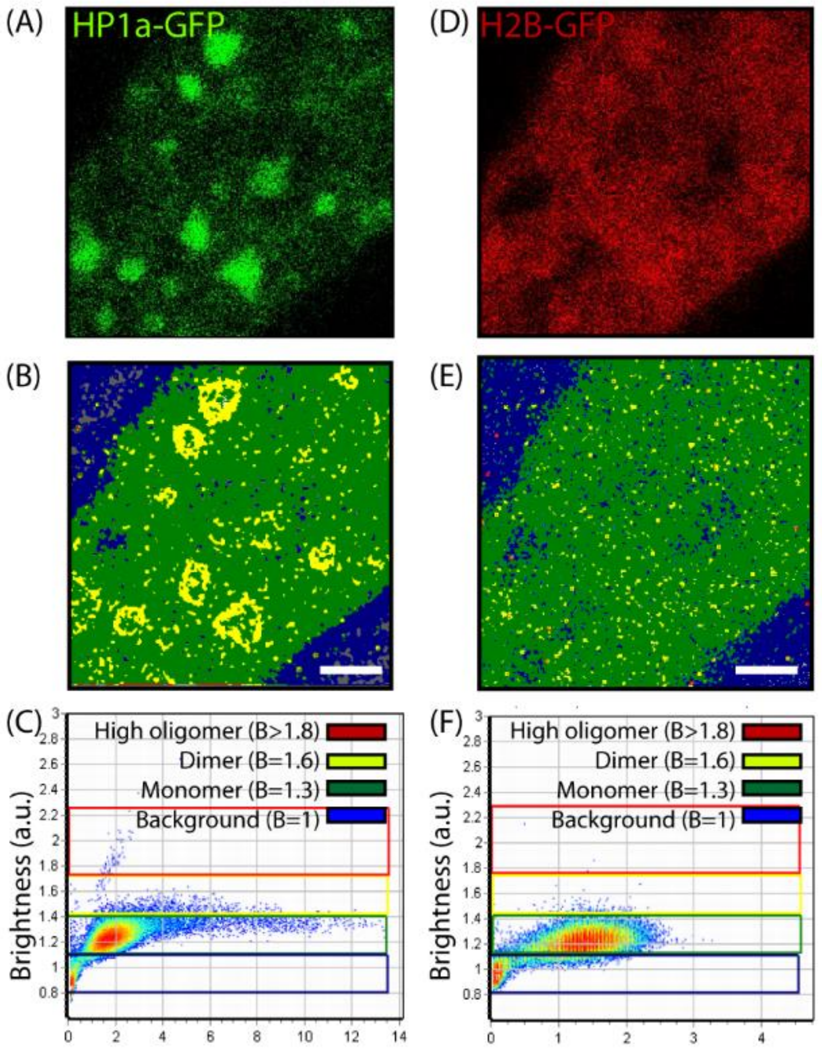


**Figure S1: Number and brightness analysis of HP1 alpha and H2B oligomerization in the absence of Hoechst 33342 staining.** (A) Intensity image of a NIH3T3 nucleus expressing HP1-α-EGFP. (B) Intensity image in (A) pseudo-colored according to the brightness distribution of HP1α in (C). As can be seen the HP1α dimers located around the periphery of the heterochromatin foci remain in the absence of Hoechst 33342 staining. (D) Intensity image of a NIH3T3 nucleus expressing H2B-mCherry. (E) Intensity image in (A) pseudo-colored according to the brightness distribution of H2B in (F). As can be seen H2B remains monomeric throughout the nucleoplasm in the absence of Hoechst 33342 staining.

## Derivation of the pair-correlation function for diffusing particles

The diffusion propagator is given by Equation 1:

$$C(r,t) = \frac{1}{(4\pi Dt)^{3/2}} \exp\left(-\frac{r^2}{4Dt}\right), \qquad (1)$$

where $C(r,t)$ can be interpreted as being proportional to the probability of finding a particle at position $r$ and time $t$ if the particle is at position 0 at time $t = 0$. The fluorescence intensity at any given time and position $\delta r$ from the origin is given by:

$$F(t,\delta r) = \kappa Q \int W(r) C(r + \delta r, t) dr , \qquad (2)$$

where it is assumed that the fluorescence is proportional to the concentration, quantum yield $Q$, excitation-emission laser power, filter combination, and the position of the particle in the profile of illumination described by $W(r)$. The pCF for two points at a distance $\delta r$ as a function of the delay time $\tau$ is calculated using the expression:

$$G(\tau,\delta r) = \frac{\langle F(t,0) \cdot F(t+\tau,\delta r)\rangle}{\langle F(t,0)\rangle \langle F(t,\delta r)\rangle} - 1 , \qquad (3)$$
